# Finding undiagnosed patients with hepatitis C infection: an application of artificial intelligence to patient claims data

**DOI:** 10.1038/s41598-020-67013-6

**Published:** 2020-06-29

**Authors:** Orla M. Doyle, Nadejda Leavitt, John A. Rigg

**Affiliations:** 1 Predictive Analytics, Real World Solutions, IQVIA, London, N1 9JY UK; 2 Predictive Analytics, Real World Solutions, IQVIA, 1 IMS Drive, Plymouth Meeting, PA USA

**Keywords:** Computational biology and bioinformatics, Predictive medicine

## Abstract

Hepatitis C virus (HCV) remains a significant public health challenge with approximately half of the infected population untreated and undiagnosed. In this retrospective study, predictive models were developed to identify undiagnosed HCV patients using longitudinal medical claims linked to prescription data from approximately ten million patients in the United States (US) between 2010 and 2016. Features capturing information on demographics, risk factors, symptoms, treatments and procedures relevant to HCV were extracted from patients’ medical history. Predictive algorithms were developed based on logistic regression, random forests, gradient boosted trees and a stacked ensemble. Descriptive analysis indicated that patients exhibited known symptoms of HCV on average 2–3 years prior to their diagnosis. The precision was at least 95% for all algorithms at low levels of recall (10%). For recall levels >50%, the stacked ensemble performed best with a precision of 97% compared with 87% for the gradient boosted trees and just 31% for the logistic regression. For context, the Center for Disease Control recommends screening in an at-risk sub-population with an estimated HCV prevalence of 2.23%. The artificial intelligence (AI) algorithm presented here has a precision which is substantially higher than the screening rates associated with recommended clinical guidelines, suggesting that AI algorithms have the potential to provide a step change in the effectiveness of HCV screening.

## Introduction

Chronic hepatitis C virus (HCV) is the leading cause of cirrhosis, liver cancer, and death from liver diseases as well as the primary indication for liver transplantation globally^1^. In the United States (US), the prevalence of HCV was reported to range between 0.6% and 1.5%^2–4^ with use of intravenous drugs^5^, blood transfusion (prior to 1992), high number of sexual partners and haemodialysis and body piercings or tattoos^5^ reported as common risk factors.

The advent of direct acting anti-virals (DAAs) has the potential to transform the HCV treatment landscape^6^ with these treatments hailed as curative. However, despite these advances, HCV remains a significant public health challenge with approximately half of the infected population untreated and undiagnosed^7^. Realising the potential benefits of novel treatments requires a reduction in the size of the undiagnosed population, coupled with early diagnosis so that patients can be treated before experiencing long term consequences of HCV infection^8^.

To improve diagnosis rates, the Centers for Disease Control and Prevention (CDC) introduced a risk-based screening programme for those who have used or are currently using intravenous drugs, have particular diagnoses (e.g. HIV infection), and were recipients of blood transfusions or organ transplants prior to 1992. In 2012, the CDC augmented the current recommendation for risk-based screening to include people who were born between 1945 and 1965^9^ which was motivated by the fact that 75% of the diagnosed HCV population were born during this period. Despite these updated recommendations, diagnosis rates have shown minimal increase and the guidelines have not been widely adopted^10^.

Artificial intelligence (AI), applied to population-based health records, has the potential to benefit risk-based screening programs such as the one cited above in several ways^11^. First, AI can help in fast-tracking the screening of potentially undiagnosed patients across a broad patient population by finding high-risk candidates. Second, AI may be able to reduce the number of false positives (i.e. people who are screened and who do not have the disease) which could lower the cost of the screening program and reduce unnecessary burden on HCV negative patients. Finally, advancing from a rules-based approach to a flexible, AI approach may be better placed to identify a heterogeneous population^12^. These benefits could provide progress towards the target adopted by the World Health Organisation (WHO) to eliminate viral hepatitis by 2030 with as few as 12 countries on-track to meet the target^13^.

AI algorithms, or more specifically machine learning algorithms^14^, “learn by example” where patient data are provided with corresponding patient outcomes to facilitate the training of an algorithm. The performance of the algorithm is then subsequently tested on an independent set of patient data. Machine learning methods hold considerable potential as they can handle high dimensional data (e.g. a large number of variables) and are sensitive to complex relationships within these data (e.g. non-linear and non-additive associations)^15^. These properties are particularly well-suited for modelling medical claims data which provide structured information on a patient’s interaction with the healthcare system. These data are collected and curated for administrative purposes and tend to have high rates of patient coverage albeit on data that may lack biologic or clinical granularity (e.g. the data captures whether a laboratory test was ordered, but not the outcome of the test)^16^. Nonetheless, medical claims represent a substantial view of the patient’s healthcare footprint and could, in some cases, be enriched by linking to other data sources such as electronic medical records or laboratory values.

There is a growing body of literature describing the application of machine learning to patient-level healthcare data and, more specifically, medical claims and prescription data. Uspenskaya-Cadoz O *et al*. applied ML algorithms to medical claims and prescription data in approximately 80 million patients to identify prodromal Alzheimer’s disease patients prior to diagnosis^17^. The authors reported precision (positive predictive value) of 58% at a recall (sensitivity) of 20% using gradient boosting trees. Razavian *et al*. used a combination of medical claims, prescription data, healthcare utilisation and laboratory data in 4.1 million patients to predict the onset of type 2 diabetes using penalised logistic regression^18^. Their approach outperformed a parsimonious model based on known risk factors. For HCV specifically, the authors of this study previously conducted a feasibility study into the use of medical history and prescription data for predicting the diagnosis of HCV with models achieving a precision of 72% at a recall of 50%^19^. Additional studies have used machine learning to focus on the challenge of predicting progression in HCV patients. Konerman *et al*. analysed approximately 11,000 HCV patients to identify those at risk of developing cirrhosis using the national Veterans Health Administration data^20^. They found that boosting survival tree-based models outperformed other methods with an area under the receiver operator characteristic curve of 0.77. While all of these studies have contrasting applications, overall they provide encouraging evidence for the role of machine learning applied to administrative healthcare data.

In this study, a suite of machine learning algorithms was developed to predict diagnosis or treatment of HCV in a universe of approximately 10 million patients using medical claims and prescription data captured between 2010 and 2016. Results were compared to a traditional statistical approach, so the potential benefits of the AI approach could be evaluated. The data were captured longitudinally in US medical claims and prescription data sources. AI introduces an opportunity to transform diagnostic screening initiatives. The dearth of relevant scientific evidence renders a full assessment of the role of AI challenging. This study contributes to the growing insight on AI healthcare applications and can support a more informed assessment on when and how to use AI for addressing under-diagnosis.

## Methods

### Data source

Data were extracted from proprietary IQVIA US longitudinal data assets. This real world data asset contains US prescription (LRx) and non-adjudicated medical claims (Dx) between January 2001 and September 2016. January 2010 was selected as the earliest date since there was a large increase data coverage from that point onwards. The LRx database includes 2 billion prescriptions per year with coverage up to 92% for the retail channel, 70% for traditional and specialty mail order, and 70% for long-term care^21^. LRx data are derived from electronic data received from pharmacies, payers, software providers and transactional clearinghouses. This information represents activities that take place during the prescription transaction and contains information regarding the product, provider, payer and geography. The Dx database receives just under 2 billion office-based electronic medical claims from office-based individual professionals, ambulatory, and general health care sites per year including patient level diagnosis and procedure information^21^. The Dx data includes fields related to patient demographics (age and gender), details on insurance used by the patient, and where the service took place (inpatient, outpatient, office, clinic, etc.).

All data remained de-identified throughout the entire data collection, linkage, and analysis process. All personal health information (PHI) were removed or encrypted by a proprietary, automated de-identification engine (ie, prior to being collected by IQVIA) obviating the requirement for informed consent. Both LRx and Dx data sets are longitudinally linked back to an anonymous patient token. This process has been certified as Health Insurance Portability and Accountability Act (HIPAA) compliant and institutional review board-exempt. IQVIA, has all requisite titles, licenses and/or rights to license these PHI for their use as permitted under applicable agreements.

### Patient selection

HCV patients were identified as those with a diagnosis code or treatment for HCV. This latter condition based on treatment is necessary given that not all diagnoses are observed in the data. The list of HCV diagnosis codes and HCV treatment products are listed in Tables S1 and S2, respectively. The index date for HCV patients was defined as a medical claim or prescription event occurring prior to the first observed date of diagnosis or treatment for HCV, ensuring only pre-diagnosed attributes were used. The lookback date for HCV patients was defined as the earliest observed event where a patient had activity in both prescription and medical claims databases. An observation window was defined as a time period between the index date and the lookback date.

Non-HCV patients were required to have no observed record for diagnosis of HCV or prescription for any HCV product. A case-control design was used; for each HCV patient, non-HCV patients were matched based on the timing of the index date and the length of the observation window of the HCV patient. Matching by index date and lookback length aimed to mitigate against two potential sample biases. First, matching by index date ensured the distribution of index dates between HCV and non-HCV patients were similar. If there were systematic differences in the distribution of index dates, biases may arise from temporal differences, such as changes in prescribing behaviour or data coverage over time. Second, matching by lookback length attempted to ensure that differences in the distribution of predictors between HCV and non-HCV patients reflected ‘genuine’ medical phenomena and did not simply arise because of differences in the length of the observation window.

Stratification criteria were applied to the patient universe to ensure that the analysis focused on a broad pool of patients with some minimal level of risk of HCV. That is, non-HCV patients were selected from the general population as those with healthcare seeking behaviour of relevance to HCV ensuring that the algorithm is focused on specific differences between HCV and non-HCV rather than a more generally learning the distinction between non-healthcare seeking vs. healthcare seeking patients. Relevance to HCV included hepatology care^5^, intravenous (IV) drug use^5^, alcohol abuse^5^, other chronic viral infections such as HIV^22^ and systemic disorders such as renal^23^ and respiratory^24^. The list of relevant events is provided in Table S3.

In addition to these stratification criteria all patients were required to meet the following selection criteria within the linked LRx and Dx dataset and were selected to ensure their eligibility for analyses:

- Have an index date between October 2015 and September 2016,
- Have at least 24 months of activity in the data set prior to index date,
- Have a valid entry for gender,

- Have an age between 20 and 79 at the index date,
- Have activity in both the prescription and medical claims data bases.

### Feature engineering

Features were created to describe patient demographics (age and gender) and medical history as captured in medical claims and prescription data. In their raw form, these data are represented using granular coding schemes such as disease classification codes (International Classification of Disease; ICD), product codes (National Drug Code; NDC) and procedure codes (Healthcare Common Procedure Coding System; HCPCS). Expert knowledge was used to identify a high level set of clinical concepts that describe risk factors (e.g. IV drug use), symptoms (e.g. jaundice), treatments (e.g. opioid use) and co-morbidities (e.g. depression) relevant to HCV. The full list of 98 clinical concepts is provided in Table S4; features are created from each of these concepts as an aggregation of diagnosis codes and/or an aggregation of drug and procedures codes. For e.g. the concept “anxiety” is represented by two features: the diagnosis codes relating to anxiety and separately the product and procedure codes relating to anxiety. Furthermore, aggregate count features were created to capture the number of events observed across categories; for e.g. count of all risk factors.

In order to exploit the longitudinal nature of the data, features were presented to the model using two metrics – the rate of occurrence (the count of events during the observation window divided by the length of the observation window) and the timing of the first occurrence (i.e. the number of days between the index date and the date of first occurrence). Overall, the total number of features available for modelling was 284.

### Statistical methods

#### Sampling

To ensure model estimation, selection and assessment were carried out independently, the data were randomly partitioned into three mutually-exclusive sets:

- A *training set* (80% of HCV patients) for model development
- A *validation set* (10% of HCV patients) for model assessment and selection
- A *test set* (10% of HCV patients) for assessing the performance of the final model in an unbiased manner

HCV patients were randomly assigned to one of the three sets; the ordering of the patients was first shuffled and then the first 80% were assigned to the training set with the subsequent 10% assigned the validation set and the remaining patients assigned to the test set. The ratio of HCV to non-HCV patients is an important consideration for ensuring that model performance is assessed in a manner that closely mirrors the distribution of HCV patients in the US population. If a model is applied to a test sample with an artificially lower sample of patients who do not have the disorder in question, then the false positive rate will also be artificially lower (Fig. S1). The prevalence of diagnosed HCV in the US population is reported to range between 0.6% and 1.5%^2–4^. To provide a conservative view of prevalence, each HCV patient was matched to 200 non-HCV patients in the validation and test sets. For the training set, a lower match rate of 1 to 50 was used, i.e. the non-HCV cohort were under-sampled to help alleviate the class imbalance problem^25^.

#### Modeling methods

Binary classifiers were developed to discriminate between HCV and non-HCV patients. Conventional parametric approaches were compared to non-parametric machine learning approaches as well as their combination. The following models were used:

- Conventional parametric methods
  - Logistic regression (LR; implemented using the ‘stats’ package in R^26^)
- **Non-parametric machine learning methods**
  - Random forest^27^ (RF; implemented using the ranger package^28^)
  - Gradient boosting trees^29^ (GBT; implemented using the xgboost package^30^)
- A stacked ensemble based on gradient boosted trees

A stacked ensemble^31^ combines output from several individual learners. A stacked ensemble has the potential of capturing different properties from different methods to form a single learner that can improve overall predictive performance. Here, a stacked ensemble was used to combine the model scores from all three methods (LR, RF and GBT). The model scores from the validation set were used as the features to train an GBT to form the stacked learner in the ensemble which was then applied to the test set.

These algorithms were chosen to incorporate a classical approach from statistical modelling (logistic regression) as well as two of the most popular tree-based approaches in machine learning –random forest and gradient boosting trees. Tree-based approaches were selected due to their ability to model non-linearities between the features and the outcome variable as well as interaction terms between features. Finally, a stacked ensemble was developed to combine the potentially complementary risk scores produced by each algorithm with the stacked ensemble providing an overall risk score for HCV.

#### Implementation, evaluation and interpretation

Algorithms were compared using a consistent data processing pipeline across techniques. LR was implemented without a regularisation penalty and applied to the features directly in the absence of any preprocessing to account for non-linearities and explicit coding of interaction terms which would require manual feature engineering available automatically in the tree-based methods. RF was implemented with a maximum of 100 individual trees and using the gini impurity to determine splits and the number of variable selected per tree as the square root of the number of available variables. GBT was implemented with a maximum of 100 individual trees using the cross-entropy loss, a maximum depth of 6 and a learning rate of 0.3.

Initial models were developed using three algorithms (LR, RF and GBT) applied to 155 features based on the demographics, aggregate counts and rate of occurrence of healthcare interactions. The best performing algorithm on these rate of occurrence features was then re-trained incorporating the temporal variables (i.e. timing of first occurrence; an additional 129 variables). Algorithms were built in this manner to provide insight into the contribution of different features metrics i.e. rate and timing of occurrences. Each model produces a score ranging from 0 to 1 for patients in the validation set, where higher scores are associated with an increased likelihood of HCV. The stacked ensemble was trained on the validation set using the model scores of these four algorithms and was then applied to the test set.

Models were evaluated using two types of performance curves, the receiver operator characteristic (ROC) curve and the precision-recall curve. The ROC curve plots the true positive rate against the false positive rate. The area under the ROC curve (AUCROC) was also calculated which ranges from 0.5 for a model performing and random to 1 for a model with perfect discriminatory performance. The precision-recall curve plots precision-recall pairs as a function of different operating points (model scores). Precision or positive predictive value (PPV) is calculated as the number of true positives divided by the sum of the number of true positives and false positives i.e. of the patients that the model identified as HCV, what percent are actual HCV patients. Recall or sensitivity is calculated as the number of true positives divided by the sum of the true positives and false negatives, i.e. of the actual HCV patients, what percent did the model identify? The precision-recall curve will be used as the primary manner of assessing model performance given that it’s well-suited to imbalanced settings, i.e. the fact that there are many more non-HCV than HCV patients^32^.

For logistic regression, the importance of features was assessed using the absolute value of the standardised regression coefficients. For RF, the gini index was used to assess feature importance. For GBT, feature importance was assessed via the average improvement in accuracy across branches due to a feature. To calculate the variable importance of the stacked ensemble, the rankings from each individual model were combined in a weighted fashion where the weighting was determined by the contribution of each individual model to the stacked ensemble.

In addition to computing feature importance, the directionality and magnitude of the effect of key features was assessed using relative risk curves. Relative risk curves show the ratio of the average model score for patients with observed feature events compared to the average model score for patients with no observed feature events.

Finally, the association was examined between model performance and the number of features used by the model using recursive feature elimination^33^. This provided insight on both the importance of dimensionality in determining predictive accuracy and the extent to which a parsimonious solution could be derived with minimal loss in performance. This exercise was carried out only for the best performing individual learner.

## Results

### Descriptive analysis

For the HCV cohort, 120,023 patients met the stratification and selection criteria. For the non-HCV cohort, approximately 60 million patients met the stratification and selection criteria. A total of 9,601,900 non-HCV patients were initially randomly selected from this pool of patients and then subsequently retained providing temporal matching criteria could be met. The non-HCV cohort was sized at approximately 10 million in accordance with known HCV prevalence^2–4^.

Table 1 provides the demographics and statistics for some of the key characteristics associated with HCV. The age and gender were similar across cohorts. For the HCV cohort, 11% were treated with HCV products (see Table S2 for a product list). A substantially higher proportion of HCV patients had a diagnosis of HIV or AIDS (5% of HCV patients vs. 1% of non-HCV patients) and/or a history of intravenous drug use (26% of HCV patients vs 3% non-HCV patients).

**Table 1:** Characteristics of the sample according the HCV status.

Variable	HCV	non-HCV
	N = 120,023	N = 9,601,900
Age (years), mean (standard deviation)	50.9 (14.2)	54.2 (14.1)
Gender (male), n (%)	53%	40%
Diagnosed with HIV or AIDS, n(%)	5.1%	0.7%
History of IV drug use, n(%)	25.9%	2.9%

**Figure 1:**
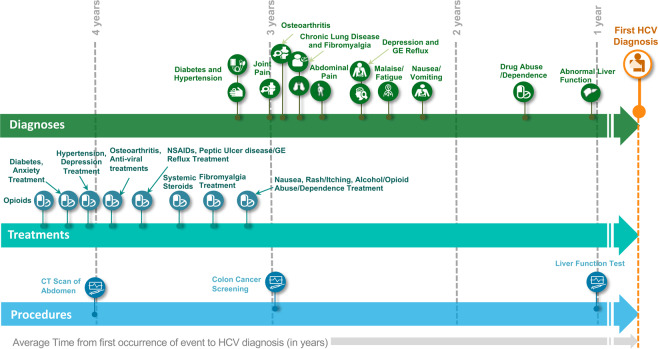
The HCV pre-diagnosis patient journey is illustrated using the average time between the date of first occurrence of events and the index date.

In Fig. 1, the HCV patient journey is illustrated using the average time between the date of first occurrence of events and the index date across HCV patients. The HCV patient journey showed that patients experienced known symptoms of HCV (joint pain, abdominal pain, malaise/fatigue, fibromyalgia) on average 2–3 years prior to their diagnosis. Treatment with NSAIDs, systemic steroids, opioids occur 3–4 years earlier indicating that these patients are seeking treatment for these symptoms prior to receiving their diagnosis for HCV. Patients are also undergoing several diagnostic test procedures close to the time of their diagnoses.

### Predictive modelling

**Figure 2:**
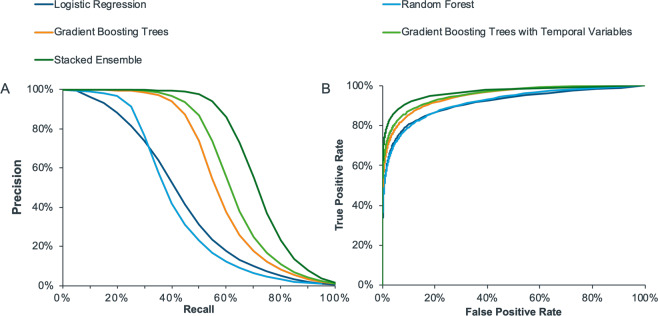
Performance curves for logistic regression, random forest and gradient boosting trees (trained on the patient demographics and rate of occurrence of clinical events), gradient boosting trees (trained on the patient demographics, rate of occurrence of clinical events and temporal variables), and the stacked ensemble (trained to combine the predictions of all four learners). Panel A displays the precision-recall curves and panel B shows the receiver operator characteristic curves. Note that recall and true positive rate are both synonymous with sensitivity.

Initially, LR, RF and GBT were developed using patient demographics and the rate of occurrence of clinical events representing 160 features in total. The predictive algorithms were developed on the training set and subsequently applied to the validation set. Performance curves are displayed in Fig. 2 in terms of both ROC and precision-recall curves. The validation AUCROC was similar for both LR and RF (0.91) but higher for GBT (0.95). If the objective was to find 10% or less of the HCV patients (i.e. 10% recall), then all three models perform similarly. However, for recall levels >30% GBT was the most performant; at 50% recall for every 100 patients identified by the model, 74 of these were true positives. For recall levels greater than 50%, the reduction in precision was steep. The GBT model was then re-trained to include features capturing the temporal nature of events with a total of 284 features with a resultant AUCROC of 0.95 on the validation sample and a corresponding training AUCROC of 0.96. Figure 2 illustrates an improvement in the precision-recall curve with the inclusion of temporal variables. Specifically, precision at 50% recall increased from 74% to 87% through temporal feature engineering.

The predictions from all four models (LR, RF, GBT with and without temporal variables) on the validation data set were then used as features to train a stacked ensemble which was subsequently applied to the test data set. The stacked ensemble achieved an AUCROC of 0.96. In terms of precision-recall curves, as before, at low levels of recall (<10%), all models performed similarly, however, the stacked ensemble outperformed all models at higher levels of recall (Fig. 2). For instance, the stacked ensemble achieved a specificity of 99.9% and precision of 97% at a recall level of 50% i.e. approximately 97 in 100 patients will be correctly identified as HCV if the objective is to find at least 50% of HCV patients (confusion matrices provided in Table S5).

In Fig. S2, the contribution of each model within the stacked ensemble is displayed. As expected the GBT including temporal variables has the largest influence on the stacked ensemble (feature importance: 76%). The LR model is the second most highly ranked (feature importance: 18%). The RF and GBT (not including temporal variables) make a negligible contribution.

### Model interpretation

**Figure 3:**
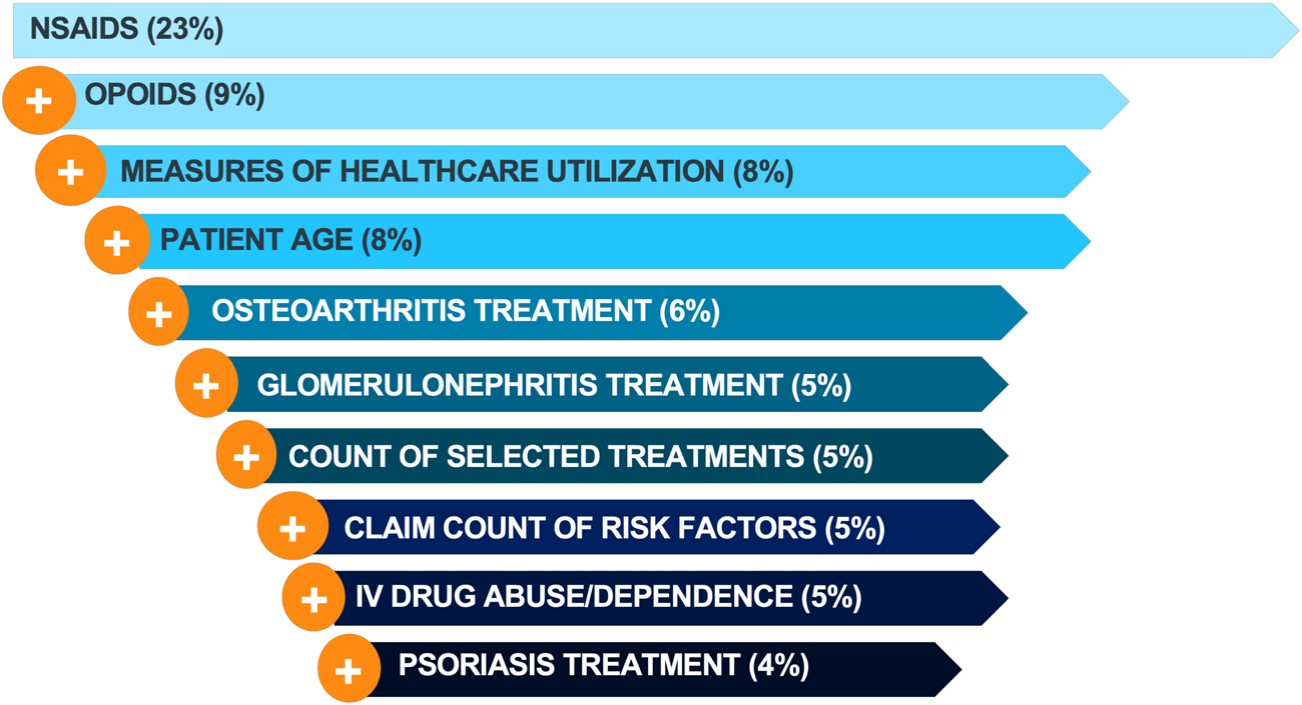
Variable importance from the stacked ensemble showing the key drivers for predicting undiagnosed HCV patients. The percentages in brackets represent the contribution of each predictor to the model performance.

In Fig. 3, the top 10 most important of the 284 variables included are listed for the stacked ensemble. Overall, age, the use of intravenous drugs and prescribed treatments were identified as key factors in discriminating between HCV and non-HCV patients. Prescriptions for non-steroidal anti-inflammatory drugs (NSAIDs) and treatments for pain both appeared in the top 10 feature importance with NSAIDs ranked as most important. Treatments relating to both osteoarthritis and rheumatoid arthritis were also highly ranked.

**Figure 4:**
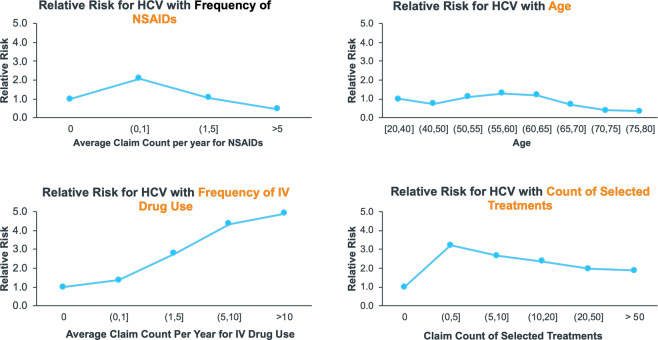
Relative Risks are displayed for some of the key variables driving prediction of HCV in the stacked ensemble.

Relative risk curves for the top three most important features and the count of unique selected treatments patients received are displayed in Fig. 4. Patients who were prescribed NSAIDs up to once per year had a two-fold increase in risk of HCV compared with patients who had not received treatment with NSAIDs whereas patients with more frequent use of NSAIDS (>1 per year) had a similar or reduced risk of HCV. A modest increase in risk was observed for patients aged between 50 and 60 years (relative risk of 1.4) as compared to patients aged between 20 and 40 years. Risk of HCV increased by five-fold in patients with 10 or more instances of IV drug use as compared to patients without evidence of IV drug use. Patients with up to five unique treatments were observed to have a three-fold increase in risk of HCV as compared to patients without these selected treatments.

**Figure 5:**
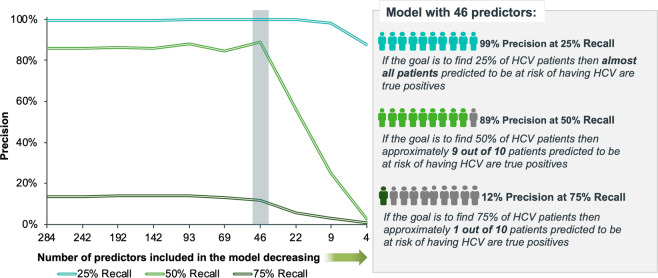
Recursive feature elimination was used to assess the impact of reducing the features on the performance as measured by precision at 25%, 50% and 75% recall.

In addition to understanding the ranking of the top variables, the relationship between performance and model complexity was assessed. In Fig. 5, the precision at three levels of recall is displayed in relation to the feature set size. This figure shows that reducing the feature set size from 284 to 46 has minimal negative impact on performance indicating that there is an opportunity to substantially reduce the model complexity without impact on model performance.

## Discussion

Tackling the considerable under-diagnosis of HCV is a key challenge in eliminating HCV. In the US, an estimated 1.8 million people remain undiagnosed with HCV^2^ with the majority of patients remaining chronically infected unless treated. In some cases, chronic infection can lead to life-threatening consequences such as cirrhosis and liver cancer. Increasing the diagnosis rate could help to reduce the period of untreated chronic infection which could help reduce the risk of progression and enable patients to access curative treatments.

In this retrospective, case-control cohort study based on US medical claims and prescription data, predictive models were developed to identify undiagnosed HCV patients. The descriptive evidence shows that patients who receive a diagnosis for HCV have substantial interaction with the health system in the four years preceding the diagnosis. First, this provides preliminary evidence that these patients could potentially be diagnosed earlier in their journey, leading to anticipated improvements in outcomes. Second, though many of these pre-diagnosis interactions are associated with well-recognised risk factors, use of a simple rules-based risk screening programme is unlikely to be highly effective given the low specificity of the risk factors. For instance, use of IV drugs was observed in just 26% of patients who received an HCV diagnosis. Compared to a simple rules-based approach, an AI/machine learning algorithm can identify complex, often subtle associations, producing potentially a far more effective means of finding undiagnosed patients.

Four types of classifiers were estimated on patients categorised as either HCV or non-HCV. For HCV patients, features were based on information prior to the earliest observed diagnosis of HCV or use of HCV-specific treatments. The learners included logistic regression, random forest, GBT (a gradient boosting tree-based model), and a stacked ensemble that combined predictions from all other learners. The use of logistic regression, a traditional choice of statistical method, alongside machine learning methods enabled a direct comparison between different modelling choices and hence a means of quantifying the potential benefit of machine learning vs. a more traditional approach.

The stacked ensemble was the best performing model producing highly encouraging results with an AUCROC of 0.96 and precision (PPV) of 97% at 50% recall (sensitivity) validated on an independent test set. In other words, at this choice of operating point, for every 100 patients flagged by the model, 97 patients were subsequently classified to have HCV. By way of a relevant benchmark, the CDC recommends that all individuals born between 1945 and 1965 should be screened for HCV; this population is estimated to have a prevalence of HCV of 2.23%. The far greater precision of the AI algorithm presented in this study suggests that AI solutions could provide a step-change in the effectiveness of HCV screening programmes.

Important risk factors identified by the algorithm included age, treatments for pain and arthritis as well as recognised HCV symptoms such as joint pain and sore muscles. IV drug use was also an important factor, associated with over a four-fold increase in the risk of HCV. The algorithm was also “stress-tested” by assessing how many low importance variables could be removed before a notable drop in performance was observed. In this case, 46 features were required to retain performance, indicating that a solution (such as a typical rules-based screening programme) that focuses on a handful of features is not sufficient to distinguish the complex pre-diagnosis footprint of HCV patients.

The evidence in this study points to the substantial potential benefit of AI in tackling problems of under-diagnosis in the context of complex disease odysseys. Notwithstanding, there are many limitations to this study, including the following:

First, this study focused on the pre-diagnosis history of HCV patients up to the point immediately prior to diagnosis or treatment. Introducing an ‘offset’ period of say 12 or 24 months, where data from the immediate period prior to HCV diagnosis or treatment is not used, would encourage the model to focus on an earlier part of the patient diagnostic journey. In turn, this approach may produce a model that is more effective at finding undiagnosed patients earlier in their journey and hence provide greater potential for improved patient health.

Second, medical claims and prescriptions were selected and aggregated by clinical experts to provide features that describe misdiagnoses, treatments, risk factors, and procedures that are relevant to HCV. This has both advantages and disadvantages. Some advantages include the ability to encode information on risk factors within the data ensuring that patients who are asymptomatic but are higher risk (e.g. patients with a history of IV drug use) are considered for screening. However, this hypothesis-driven approach is highly time-consuming and manual in nature and can lead to under-performance since potentially important features may be omitted. In general, combining domain knowledge with a data-driven approach would be expected to lead to notable improvements in performance^34,35^.

Third, an external prospective validation of this algorithm would substantially enhance the scientific credibility of this algorithm under test conditions that are a highly accurate representative of the deployment setting.

Fourth, medical claims data under-represent patients with restricted access to healthcare. To the extent that these patients disproportionally comprise undiagnosed HCV patients, this could introduce a sample bias and hence limit the applicability of any medical claims-based model applied to a representative sample of the population. Additionally, the imbalanced nature of the panel, along with gaps in data coverage (many of which would not be expected to be missing at random), could lead to biased results such as the under-representation of important risk factors^36^.

Fifth, some people with HCV in this study will not receive either an HCV diagnosis or treatment within the observation window. These patients will be erroneously classified in this study as non-HCV. Thus, there is a known misclassification problem. However, it is likely that this problem introduces only a relatively modest amount of noise given that it would be expected that the undiagnosed HCV patients would comprise a small portion of the so-called non-HCV cohort.

In light of these considerations, the overarching conclusion is encouraging with AI solutions having the potential to transform HCV case-finding and provide progress towards the WHO goal of eliminating viral hepatitis by 2030^13^. There are multiple options for deploying this solution in the field ranging from those which are “live” today and those that require considerable interdisciplinary development prior to deployment. Solutions that are in live deployment today are typically those that support physician targeting by the commercial function of life science companies whereby high risk HCV patients can be linked to their treating physician who then becomes a high value target for a variety of personal and non-personal out-reach activities. The use case within life sciences companies is now broadening to consider the use of these solutions in clinical trial recruitment. Beyond the life sciences sector, the potential for AI disease detection applications is exciting and far-reaching. In principal, these algorithms can be deployed in routine clinical practice. At-risk patients could be flagged when they present via a pop-up at the point-of-care. Alternatively, the algorithms could run off-line and high risk patients could be flagged to the practice manager. Information could be provided on relevant diagnostic screening programs, endorsed by professional clinical associations, which the practice could leverage to promote early diagnosis. The prospect is unquestionably exciting. But there are many hurdles which need to be overcome ranging from infrastructure to interoperability to information governance to clinical adoption^37^. The call to arms is now directed towards overcoming the other many hurdles so that AI can realise its full potential in clinical practice, finding undiagnosed patients in a timely manner and improving patient health. The value of this study is to establish the potential effectiveness of an AI-focused approach based on routinely collected health data.

## Supplementary information

Supplementary information.

## Data Availability

The datasets generated during and/or analysed during the current study are not openly available as the underlying data sources are used for commercial purposes by IQVIA.
