# Supplementary material for: Finding undiagnosed patients with hepatitis C infection: an application of artificial intelligence to patient claims data
Source: Sci Rep. 2020 Jun 29;10:10521. doi: 10.1038/s41598-020-67013-6 (PMC7324575; doi:10.1038/s41598-020-67013-6)
Supplement: Supplementary file 1 — Supplementary information. [file 41598_2020_67013_MOESM1_ESM.docx]

Finding undiagnosed patients with hepatitis C infection: an application of artificial intelligence to patient claims data

Supplementary Information

Orla M. Doyle^1^, Nadejda Leavitt^2^ and John A. Rigg^1^.

^1^ Predictive Analytics, Real World Solutions (RWS) Technology, IQVIA, London, N1 9JY, UK.

^2^ Predictive Analytics, Real World Solutions (RWS) Technology, IQVIA, 1 IMS Drive, Plymouth Meeting, PA, USA.

## Diagnosis codes and treatment products for HCV

| **Diagnosis Code** | **ICD** | **Description** |
| --- | --- | --- |
| 070.41 | ICD-9 | ACUTE HEPATITIS C WITH HEPATIC COMA |
| 070.44 | ICD-9 | CHRONIC HEPATITIS C WITH HEPATIC COMA |
| 070.51 | ICD-9 | ACUTE HEPATITIS C WITHOUT MENTION OF HEPATIC COMA |
| 070.54 | ICD-9 | CHRONIC HEPATITIS C WITHOUT MENTION OF HEPATIC COMA |
| 070.7 | ICD-9 | UNSPECIFIED VIRAL HEPATITIS C |
| 070.70 | ICD-9 | UNSPECIFIED VIRAL HEPATITIS C WITHOUT HEPATIC COMA |
| 070.71 | ICD-9 | UNSPECIFIED VIRAL HEPATITIS C WITH HEPATIC COMA |
| B17.10 | ICD-10 | ACUTE HEPATITIS C WITHOUT HEPATIC COMA |
| B17.11 | ICD-10 | ACUTE HEPATITIS C WITH HEPATIC COMA |
| B18.2 | ICD-10 | CHRONIC VIRAL HEPATITIS C |
| B19.20 | ICD-10 | UNSPECIFIED VIRAL HEPATITIS C WITHOUT HEPATIC COMA |
| B19.21 | ICD-10 | UNSPECIFIED VIRAL HEPATITIS C WITH HEPATIC COMA |
| K73.0 | ICD-10 | CHRONIC PERSISTENT HEPATITIS, NOT ELSEWHERE CLASSIFIED |
| V02.62 | ICD-10 | CARRIER OR SUSPECTED CARRIER OF HEPATITIS C |
| V12.09 | ICD-10 | PERSONAL HISTORY OF OTHER SPECIFIED INFECTIOUS AND PARASITIC DISEASE |
| Z22.52 | ICD-10 | CARRIER OF VIRAL HEPATITIS C |
| Z86.19 | ICD-10 | PERSONAL HISTORY OF OTHER INFECTIOUS AND PARASITIC DISEASES |

Table S 1 List of ICD-9 and ICD-10 codes used to select HCV patients.

| **Treatments** |
| --- |
| DAKLINZA |
| EPCLUSA |
| HARVONI |
| INCIVEK |
| INFERGEN |
| MODERIBA |
| OLYSIO |
| PEGASYS PROCLICK |
| PEGINTRON REDIPEN |
| REBETOL |
| REBETRON 1000 |
| REBETRON 1000-PEN |
| REBETRON 1200 |
| REBETRON 1200-PEN |
| REBETRON 600 |
| REBETRON 600-PEN |
| RIBAPAK |
| RIBASPHERE |
| RIBASPHERE RIBAPAK |
| RIBATAB |
| RIBAVIRIN |
| SOVALDI |
| TECHNIVIE |
| VICTRELIS |
| VIEKIRA PAK |
| VIEKIRA XR |
| VIRAZOLE |
| ZEPATIER |

Table S 2 List of products used to define treatment for HCV.

## Stratification criteria

The complete list of stratification criteria are contained in table below.

| Abnormal liver function tests |
| --- |
| Alcohol Abuse |
| Anxiety |
| Chronic Kidney Disease |
| Chronic Lung Disease |
| Convulsions |
| Depression |
| Dyspnea |
| Edema |
| Fever |
| Fibromyalgia |
| Hepatitis B |
| HIV or AIDs |
| Insomnia |
| IV Drug Use |
| Other chronic liver disease |
| Thrombocytopenia |

Table S 3 Complete list of stratification criteria.

## List of Clinical Concepts for Feature Engineering

| **PREDICTORS - CLINICAL CONCEPTS** |
| --- |
| AGE |
| GENDER |
| ABDOMINAL PAIN |
| ABNORMAL BOWEL MOVEMENTS |
| ABNORMAL LIVER FUNCTION TESTS |
| ALCOHOL ABUSE |
| ALCOHOL OPIOID ABUSE |
| ALCOHOLIC LIVER DISEASE |
| ALOPECIA AREATA |
| ANALGESICS |
| ANTIPHOSPHOLIPID SYNDROME |
| ANXIETY |
| ASCITES |
| AUTOIMMUNE HEMOLYTIC ANEMIA |
| BRUISING |
| CANCER |
| CHEMISTRY |
| CHOLANGITIS |
| CHRONIC LUNG DISEASE |
| CHRONIC FATIGUE SYNDROME |
| CHRONIC KIDNEY DISEASE |
| CYTOMEGALOVIRUS |
| COLITIS |
| COLON CA SCREENING |
| CONFUSION |
| CONVULSIONS |
| CRYOGLOBULINEMIA |
| CT OF ABDOMEN |
| DARK URINE |
| DEPRESSION |
| DIABETES |
| DROWSINESS |
| NSAIDS |
| DYSPNEA |
| EDEMA |
| EXPOSURE TO HAZARDOUS FLUIDS |
| FEVER |
| FIBROMYALGIA |
| FLU |
| GASTROENTEROLOGIST CLAIM COUNT |
| GE REFLUX |
| GLOMERULONEPHRITIS |
| HEADACHE |
| HEARTBURN |
| HEMATOLOGIST CLAIM COUNT |
| HEMOCHROMATOSIS |
| HEMODIALYSIS |
| HEMOPHILIA |
| HEMORRHOIDS |
| HEPATIC ENCEPHALOPATHY |
| HEPATITIS B |
| HEPATOCELLULAR CARCINOMA |
| HEPATOLOGIST CLAIM COUNT |
| HIGH RISK SEXUAL BEHAVIOR |
| HIV OR AIDS |
| HYPERLIPIDEMIA |
| HYPERTENSION |
| HYPOTHYROIDISM |
| IRRITABLE BOWEL SYNDROME |
| INFECTIOUS DISEASE SPECIALIST CLAIM COUNT |
| INSOMNIA |
| IDIOPATHIC PULMONARY FIBROSIS |
| ITCHING |
| INTAVENOUS DRUG USE |
| JAUNDICE |
| JOINT PAIN |
| LEG SWELLING |
| LICHEN PLANUS |
| LOSS OF APPETITE |
| LUPUS |
| MALAISE/FATIGUE |
| MILK ALLERGY |
| NAUSEA |
| NON-HODGKIN'S LYMPHOMA |
| NON-ALCOHOLIC STEATOHEPATITIS |
| NONINFECTIOUS HEPATITIS |
| OBESITY |
| ORGAN TRANSPLANT |
| OSTEOARTHRITIS |
| OTHER CHRONIC LIVER DISEASE |
| PAIN |
| PEPTIC ULCER DISEASE |
| PORPHYRIA CUTANEA TARDA |
| PROSTATE CANCER |
| PSORIASIS |
| RASH |
| REACTIVE ARTHRITIS |
| RENAL CANCER |
| RHEUMATOID ARTHRITIS |
| SJOGRENS DISEASE |
| SLURRED SPEECH |
| SPIDER ANGIOMAS |
| SEXUALLY TRANSMITTED DISEASES |
| STEROIDS SYSTEMIC |
| THROMBOCYTOPENIA |
| THROMBOSIS |
| URINARY RETENTION |
| VASCULITIS |
| VITILIGO |
| WEIGHT LOSS |

Table S 4 List of clinical concepts used for creating features for the algorithm. In some case a single clinical concept was used to derive two features: one capturing the activity recorded using diagnoses codes and another capturing the treatment activity via product codes or procedure codes.

## Model validation and the impact of the size of the control cohort


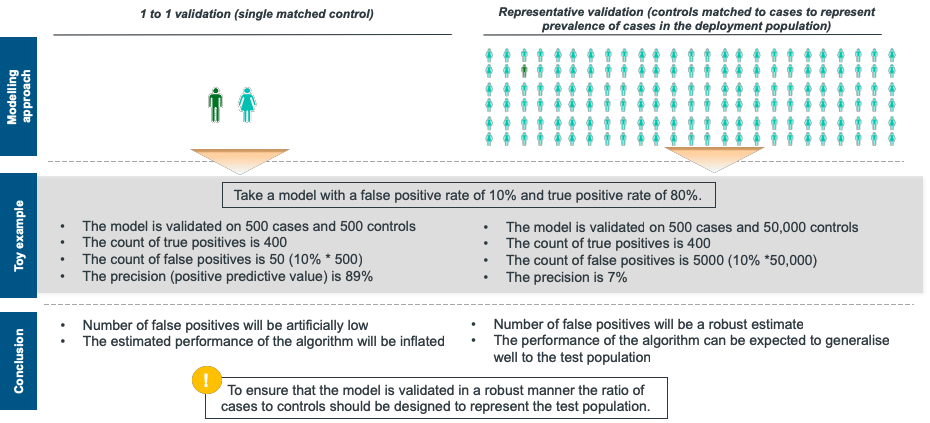


Figure S1 Toy example illustrating the substantial effect on testing the model on a population that represents the deployment population in terms of expected ratio of positive to negative cases.

The ratio of HCV to non-HCV patients is an important consideration for ensuring that model performance is assessed in a manner that closely mirrors the distribution of HCV patients in the US population. If a model is applied to a test sample with an artificially lower sample of patients who do not have the disorder in question, then the false positive rate will also be artificially lower. In Figure S1, the model performance is shown for two settings – (i) an extreme case where for each positive case we only have one negative case (i.e. one to one ratio between positive and negative patients) which represents a highly mismatched setting as there are likely to be far more negative cases than positive cases in virtaully all disease detection scenarios and (ii) a representative case where the ratio of positive to negative cases resembles the expected ratio in the known population. This toy example shows that model performance metrics are highly inflated when a substantially lower pool of negative cases are used in testing.

## Confusion matrices for the stacked ensemble model

Confusion matrices were generated for the stacked ensemble model at two levels of recall: 50% and 75%.

|  |  | **Predicted @ 50% recall** | |
| --- | --- | --- | --- |
|  |  | HCV | non-HCV |
| **Actual** | HCV | 5,870 | 5,886 |
|  | non-HCV | 208 | 2,305,317 |
|  |  |  |  |
|  |  | **Predicted @ 75% recall** | |
|  |  | HCV | non-HCV |
| **Actual** | HCV | 8,815 | 2,941 |
|  | non-HCV | 21,132 | 2,284,393 |

Table S 5 Confusion matrices. The top left value is the count of trues positives, the top right value is the count of false negatives, the bottom right value is the count of false positives and the bottom right value is the count of true negatives

## Contribution of the individual learners to the stacked ensemble


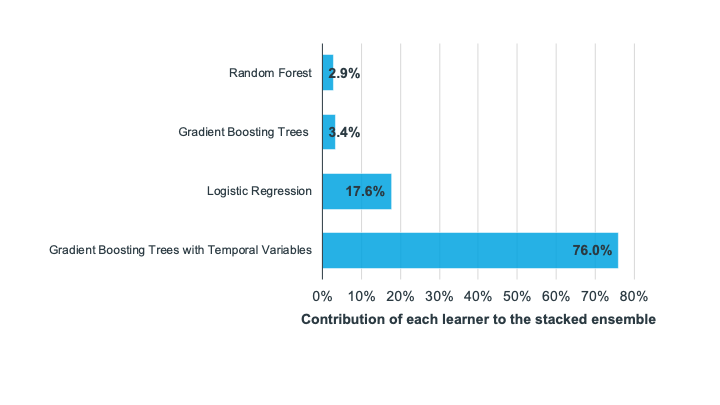


Figure S2 Percentage contribution of individual learners (Random Forest, GBT, Logistic Regression, GBT with temporal variables) to the stacked ensemble.
